# Prognostic role of the preoperative neutrophil-to-lymphocyte ratio and albumin for 30-day mortality in patients with postoperative acute pulmonary embolism

**DOI:** 10.1186/s12890-020-01216-5

**Published:** 2020-06-24

**Authors:** Chuan Liu, Hui-lu Zhan, Zhang-Heng Huang, Chuan Hu, Yue-Xin Tong, Zhi-Yi Fan, Meng-Ying Zheng, Cheng-Liang Zhao, Gui-Yun Ma

**Affiliations:** 1 Department of Orthopedic, Affiliated Hospital of Chengde Medical University, Chengde, China; 2 Graduate School of China Medical University, Shenyang, China; 3 School and Hospital of Stomatology, Wenzhou Medical University, Wenzhou, China; 4 Qingdao University medical college, Qingdao, China; 5 Second Clinical Medical College of Wenzhou Medical University, Wenzhou, China

**Keywords:** Neutrophil-to-lymphocyte ratio, Albumin, Mortality, Postoperative acute pulmonary embolism

## Abstract

**Background:**

This retrospective study aimed to investigate the prognostic value of the neutrophil-to-lymphocyte ratio (NLR) and albumin for 30-day mortality in patients with postoperative acute pulmonary embolism (PAPE).

**Methods:**

We retrospectively reviewed the medical records of 101 patients with PAPE admitted from September 1, 2012, to March 31, 2019. The characteristics, surgical information, admission examination data and mortality within 30 days after PAPE were obtained from our electronic medical recording system and follow-up. The associations between the NLR, PLR, and other predictors and 30-day mortality were analyzed with univariate and multivariate analyses. Then, the nomogram including the independent predictors was established and evaluated.

**Results:**

Twenty-four patients died within 30 days, corresponding to a 30-day mortality rate of 23.8%. The results of the multivariate analysis indicated that both the NLR and albumin were independent predictors for 30-day mortality in patients with PAPE. The probability of death increased by approximately 17.1% (OR = 1.171, 95% CI: 1.073–1.277, *P* = 0.000) with a one-unit increase in the NLR, and the probability of death decreased by approximately 15.4% (OR = 0.846, 95% CI: 0.762c–0.939, *P* = 0.002) with a one-unit increase in albumin. The area under the curve of the nomogram was 0.888 (95% CI: 0.812–0.964).

**Conclusion:**

Our findings showed that an elevated NLR and decreased albumin were related to poor prognosis in patients with PAPE. The NLR and albumin were independent prognostic factors for PAPE.

## Background

Postoperative acute pulmonary embolism (PAPE) is one of the most dangerous complications following operations, with an incidence between 0.9 and 3.1% [1–4]. Although the methods of diagnosis and treatment of PAPE have been continuously developed in recent years, including imaging diagnosis, interventional surgery and medicinal chemotherapy, the overall survival rate of patients with PAPE is extremely low. It was reported that the short-term mortality of patients with PAPE was between 10 and 23.1% [5–9]. Therefore, identifying the preoperative risk factors associated with mortality may help to direct more aggressive treatment strategies, such as fibrinolytic therapy, towards patients who will derive the greatest benefit.

The mechanism of inflammatory reactions is closely related to the occurrence and development of thromboembolism [10]. In recent years, many researchers have reported that some predictors based on inflammation are associated with prognosis in patients with pulmonary embolism, such as the neutrophil-to-lymphocyte ratio (NLR), monocyte-to-lymphocyte ratio (MLR), red blood cell distribution width (RDW), and C-reactive protein (CRP) [11–14]. In addition, the relationship between nutritional status and prognosis in patients with pulmonary embolism has also been extensively studied in a previous study [15]. Plasma albumin is one of the important indicators reflecting systemic nutritional status and is associated with prognosis in patients with acute pulmonary embolism [16]. However, previous studies have focused on nonsurgical patients, and no studies have focused on the relationship between these predictors and mortality in patients with PAPE. Therefore, this study was performed to investigate the relationship of admission NLR, plasma albumin and other predictors with 30-day mortality in patients with PAPE.

## Methods

### Patients

We performed a single-center, retrospective and case-control study. The medical records of consecutive patients who were diagnosed with pulmonary embolism from September 1, 2012, to March 31, 2019, in our hospital were reviewed, and patients who were diagnosed with acute pulmonary embolism within 90 days postoperatively were included in this study. In the present study, only patients with pulmonary embolism confirmed by computed tomography pulmonary angiography (CTPA) were defined as pulmonary embolism patients, and patients with suspected but unconfirmed pulmonary embolism by examination were not defined as pulmonary embolism patients. Patients were excluded if they underwent cardiac surgery, did not have complete data, had received blood transfusion within 1 month preoperatively, had comorbid infection, and had comorbid hematological disease or received immunosuppressive therapy within 1 month preoperatively. Finally, 101 patients met our inclusion criteria. All patients with PAPE met the diagnostic criteria of the 2014 ESC guidelines on the diagnosis and management of acute pulmonary embolism [17].

### Data collection

Data were extracted from the hospital electronic database by two independent doctors. If controversial data were encountered, the two doctors who collected the data underwent discussion to reach an agreement. All patients’ characteristics (sex, age, BMI, smoking history and drinking history), comorbidities (hypertension, diabetes, respiratory diseases, chronic coronary heart disease, chronic arrhythmia, history of stroke and chronic renal failure), surgical information (surgical type and ASA level), admission examination data (neutrophil-to-lymphocyte ratio; platelet-to-lymphocyte ratio; monocyte-to-lymphocyte ratio; hemoglobin; white blood cell; platelet; mean platelet volume; platelet distribution width; red cell distribution width; glucose; neutrophil; lymphocyte; creatinine and albumin) and the situation within 30 days after PAPE were obtained through our electronic medical recording system and follow-up. Complete blood counts (CBCs), blood glucose levels, and albumin assessments were carried out at the biochemistry laboratory of our hospital. The NLR was obtained by dividing the absolute neutrophil counts by the absolute lymphocyte counts from the same blood sample, the PLR was obtained by dividing the absolute platelet counts by the absolute lymphocyte counts from the same blood sample, and the MLR was obtained by dividing the absolute monocyte counts by the absolute lymphocyte counts from the same blood samples. All test results were obtained from the same blood sample test within 3 days before surgery.

### Statistical analysis

Data analysis was performed using the Statistical Package for the Social Sciences version 25.0 for Windows (IBM, Chicago, IL, USA). Data with a normal distribution are represented as the mean ± standard deviation, and Student’s t-test was used to compare two groups. The data with an abnormal distribution are represented as medians (interquartile ranges), and the Mann-Whitney U test was used to compare two groups. Categorical variables are represented as numbers or percentages, and the χ^2^ test or Fisher’s exact test was performed for categorical variables. Based on the univariate analysis, variables with a *P* value < 0.05 were included in the multivariate logistic regression analysis to confirm the independent risk factors. Forward logistic regression analysis was conducted to estimate the OR and 95% CI for 30-day mortality of the NLR, albumin and other parameters after adjusting for potential confounding factors. The receiver operating characteristic (ROC) curve was used to examine the performance of independent risk factors in predicting 30-day mortality. The area under the curve (AUC) was derived from the ROC curve, which ranged from 0.5 to 1.0 – with higher values indicating higher discriminatory ability, and the Youden Index (maximum [sensitivity +specificity] minus 1) was adopted to define the optimal cut-off value. Afterwards, a nomogram based on the independent predictors was established, and the calibration curve and decision curve analysis (DCA) were generated to evaluate the nomogram. In addition, the AUC of the nomogram was calculated, and the differences in the AUC between the nomogram and independent predictors were compared by the pROC package in R software (version 3.6.1). All *P* values < 0.05 were accepted as statistically significant.

## Results

### Baseline

During the study period, 125 patients were diagnosed with pulmonary embolism within 90 days after noncardiac surgery. Twenty-four patients were excluded because they did not meet our criteria for hematological disease (2 cases), received blood transfusion within 1 month preoperatively (13 cases), had an infection (2 cases), received immunosuppressive therapy within 1 month preoperatively (1 case) and had missing data (6 cases). Finally, 101 patients with PAPE following noncardiac surgery met our inclusion criteria and were included in this study, which included 41 males and 60 females, and the median age was 64 years (interquartile range: 57.50–71.00 years). For 101 patients, the median time of PAPE was 3 days (interquartile range: 1–5 days). Seven patients were diagnosed with massive pulmonary embolism: ten patients received fibrinolytic therapy, and the remaining patients received anticoagulant therapy. The demographic data and clinical data of deaths and survivors are listed in Table 1.

**Table 1:** Comparison of baseline and comorbidities between deaths and survivors

	Total(*n* = 101)	Deaths(*n* = 24)	Survivors(*n* = 77)	*P*
Age, yr	64.00 (57.50–71.00)^a^	67.00 (59.00–75.75) ^a^	63.00 (57.00–70.00) ^a^	0.200
Sex (Female)	60	12	48	0.283
BMI, kg/m^2^	25.85 ± 3.68	24.54 ± 4.10	26.31 ± 3.59	0.050
Smoking history	23	6	17	0.766
Drinking history	19	4	15	0.758
Surgical type				0.861
Musculoskeletal	45	12	33	
Abdominal	28	7	21	
Respiratory	10	2	8	
Gynecologic	8	2	6	
Neurosurgery	5	1	4	
Vascular	5	0	5	
ASA				0.787
II	65	16	49	
III	36	8	28	
Admission SBP	120.00 (135.00,150.00)^a^	124.50 (133.50,149.25)^a^	120.00 (136.00,151.00)^a^	0.873
Comorbidities				
Hypertension	38	9	29	0.989
Diabetes	15	2	13	0.484
Coronary heart disease	19	4	15	0.993
Respiratory diseases	9	1	8	0.600
Arrhythmia	4	1	3	1.000^b^
History of stroke	7	2	5	1.000
Renal failure	1	1	0	0.238^b^

^a^Interquartile range

^b^Fisher’s Exact test

*BMI* Body mass index, *SBP* Systolic blood pressure

### Prognostic factors of PAPE

Twenty-four patients died within 30 days, corresponding to a 30-day mortality rate of 23.8%. There were no significant differences in terms of age, sex, BMI, smoking history, drinking history, admission systolic blood pressure, surgical type or ASA level (all *P* values>0.05). There were no significant differences in terms of hypertension, diabetes, respiratory diseases, coronary heart disease, arrhythmia, history of stroke or renal failure (all P values>0.05). The baseline characteristics and comorbidities of the patients are shown in Table 1. The preoperative laboratory parameters are presented in Table 2. The NLR, neutrophil count and creatinine level were significantly higher in those who died than in survivors with PAPE (all *P* values < 0.05), and the albumin level was significantly lower in those who died than in survivors after PAPE (*P* = 0.008). There were no significant differences in the other parameters included in our research (Table 2).

**Table 2:** Comparison of admission laboratory data between deaths and survivors

	Deaths(*n* = 24)^*^	Survivors(*n* = 77)^*^	*P*
NLR	14.13 (7.67–23.04)	5.93 (2.60–8.70)	0.000
PLR	230.00 (102.25–396.24)	157.29 (103.99–243.52)	0.193
MLR	0.80 (0.44–1.27)	0.44 (0.31–0.77)	0.004
WBC, × 10^9^/L	13.84 (8.80–17.47)	9.74 (6.62–12.71)	0.001
Neutrophil, ×10^9^/L	12.21 (8.04–15.85)	6.20 (3.98–9.91)	0.000
Lymphocyte, ×10^9^/L	0.82 (0.51–1.59)	1.21 (0.90–1.82)	0.017
Monocyte, ×10^9^/L	0.63 (0.36–0.89)	0.63 (0.45–0.99)	0.621
PLT, ×10^9^/L	163.50 (131.25–215.25)	199.00 (141.00–247.00)	0.273
MPV, fL	10.15 (9.30–10.78)	10.10 (9.40–10.88)	0.631
PDW	12.75 (11.28–15.80)	13.25 (10.73–16.08)	0.990
Hb, g/L	121.00 (108.25–141.50)	108.00 (90.00–119.00)	0.795
RDW	13.15 (12.73–15.33)	13.20 (12.45–14.35)	0.369
Creatinine, μmol/L	84.40 (71.72–106.50)	72.00 (59.75–87.00)	0.023
GLU, mmol/L	8.19 (6.95–13.83)	7.60 (5.56–9.92)	0.054
Albumin, g/L	32.33 (27.37–35.69)	38.20 (32.49–45.80)	0.008

*All variables are describe as median and interquartile range

*NLR* neutrophil-to-lymphocyte ratio, *PLR* platelet to lymphocyte ratio, *WBC* white blood cell, *MLR* monocyte-to-lymphocyte ratio, *PLT* platelet, *MPV* mean platelet volume, *PDW* platelet distribution width, *Hb* hemoglobin, *RDW* red cell distribution width, *GLU* glucose

To further confirm the independent risk factors for mortality after PAPE, multivariate logistic analysis was performed. The NLR, MLR, WBC, neutrophil, lymphocyte, creatinine, and albumin (all *P* values<0.05) were included in the multivariate analysis, and the results indicated that both the NLR and albumin were independent predictors of 30-day mortality in patients with PAPE. The probability of death increased by approximately 17.1% (OR = 1.171, 95% CI: 1.073–1.277, *P* = 0.000) with a one-unit increase in the NLR, and the probability of death decreased by approximately 15.4% (OR = 0.846, 95% CI: 0.762c–0.939, *P* = 0.002) with a one-unit increase in albumin (Table 3). In addition, the results indicated that creatinine, the MLR, neutrophil, lymphocyte and WBC were no longer independent predictors in multivariate analysis (all *P* values > 0.05).

**Table 3:** Multivariate regression results of 30-days mortality

	B	SE	Wald	OR	95% CI	*P*
Albumin	−0.167	0.053	9.881	0.846	0.762–0.939	0.002
NLR	0.158	0.044	12.644	1.171	1.073–1.277	0.000

*NLR* neutrophil-to-lymphocyte ratio

### Development of a nomogram

**Fig. 1:**
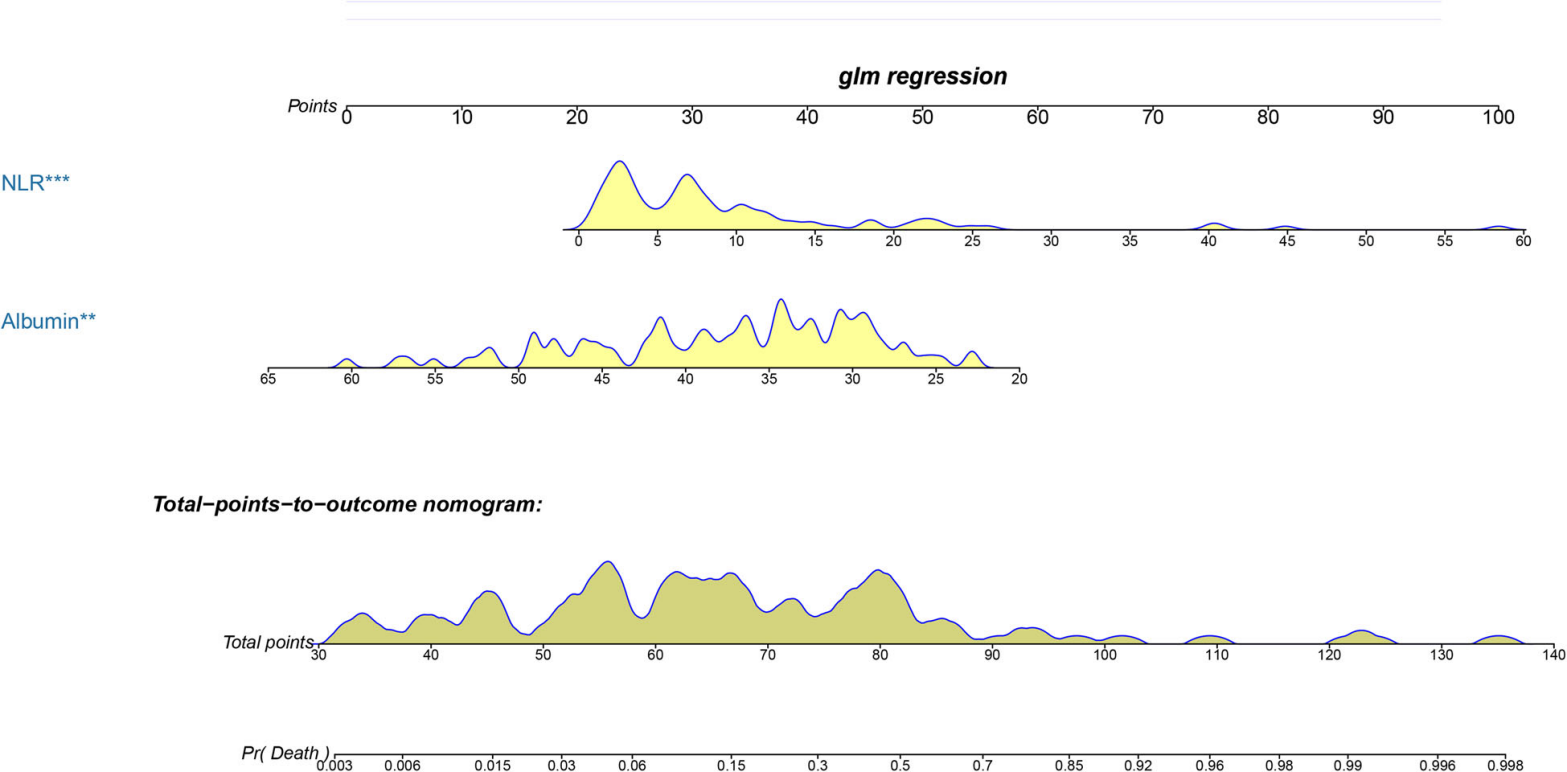
A nomogram incorporating the NLR and albumin for predicting 30-day mortality in patients with postoperative acute pulmonary embolism

**Fig. 2:**
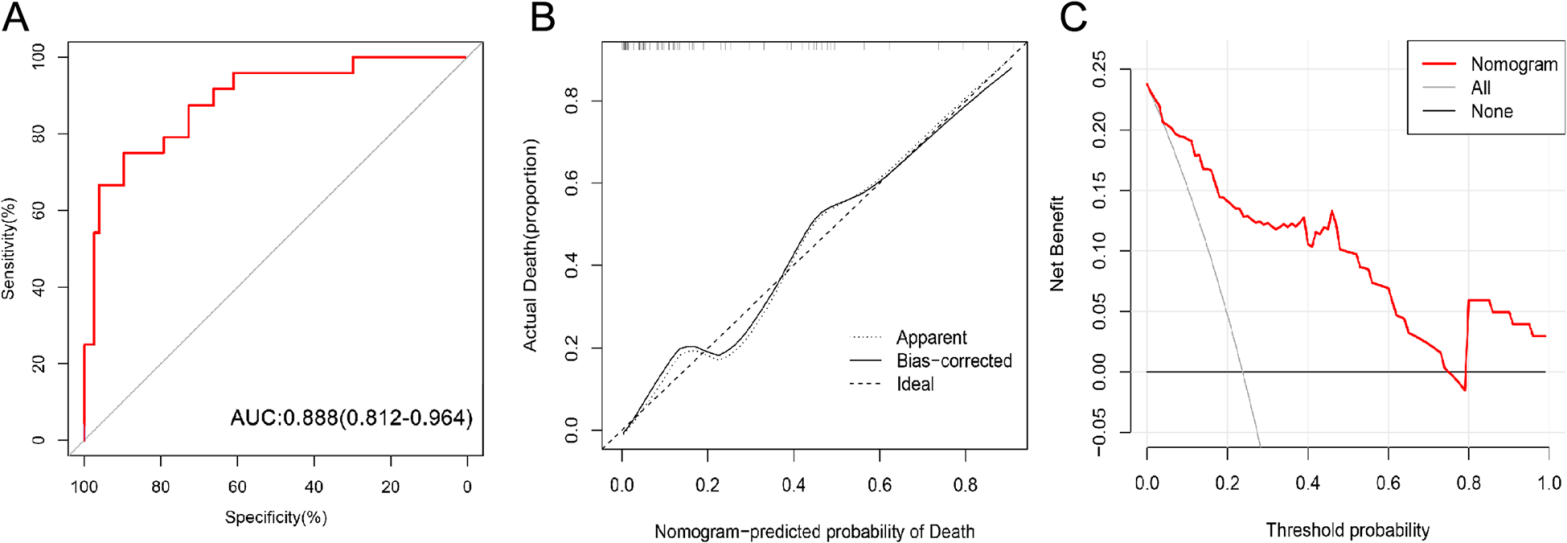
Receiver operating characteristic curve, calibration curve, and decision curve analysis of the nomogram. **a**. The area under the receiver operating characteristic curve was 0.888 (95% CI: 0.812–0.964); **b**. The calibration curve showed that the nomogram-predicted probability of death was highly consistent with the actual probability of death; **c**. The decision curve analysis indicated that if the threshold probability of a patient and a doctor was between 5 and 75%, this nomogram predicted 30-day mortality with more benefit than the scheme

**Fig. 3:**
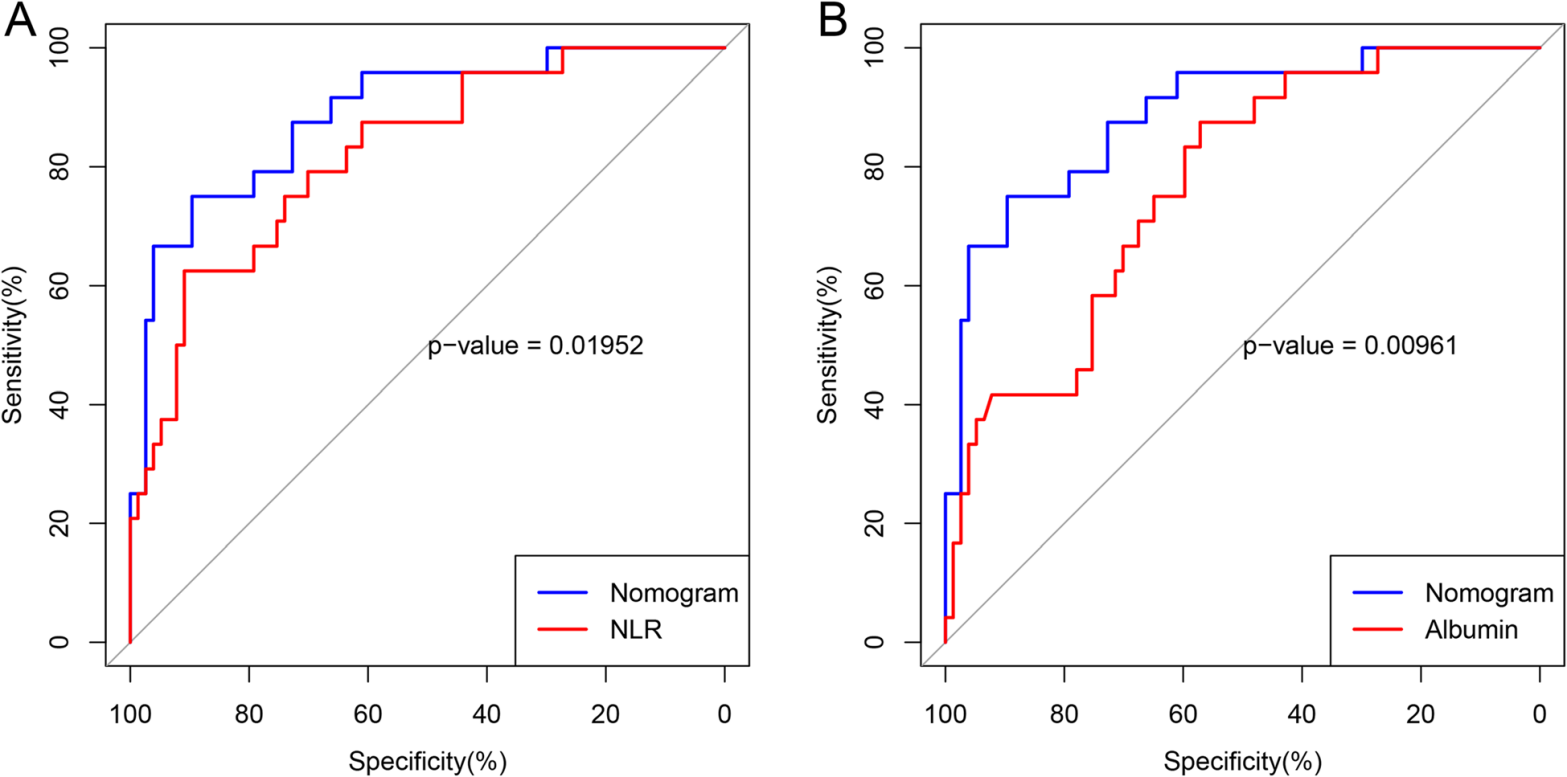
Receiver operating characteristic curve of the nomogram, the NLR, and albumin. **a**. The area under the receiver operating characteristic curve of the nomogram was significantly higher than the area under the receiver operating characteristic curve of the NLR. **b**. The area under the receiver operating characteristic curve of the nomogram was significantly higher than the area under the receiver operating characteristic curve of albumin

Based on the independent predictors, a nomogram was established to predict 30-day mortality in PAPE patients (Fig. 1). The AUC of the nomogram was 0.888 (95% CI: 0.812–0.964), which was significantly higher than that of any single predictor (*P* value< 0.05) (Table 4 and Figs. 2 and 3a). Moreover, the calibration curve is shown in Fig. 2b, and the results indicated that the prediction by the nomogram was highly consistent with the actual observations. In addition, the DCA indicated that if the threshold probability of a patient and a doctor was between 5 and 75%, this nomogram predicted 30-day mortality with more benefit than the scheme(Fig. 2c).

**Table 4:** Values of predicators in predicting 30-days mortality

Predicators	AUC	95% CI for AUC	P	Cut-off	Sensitivity	Specificity
NLR	0.823	0.729–0.917	0.000	12.00	0.625	0.909
Albumin	0.768	0.668–0.868	0.000	36.66	0.571	0.875

*AUC* area under the curve, *NLR* neutrophil-to-lymphocyte ratio

## Discussion

PAPE is one of the most dangerous complications following operations, and it is necessary to predict the prognosis of patients early. To the best of our knowledge, this is the first study to investigate predictors and establish a nomogram of 30-day mortality among patients with PAPE following noncardiac surgery. The primary findings of our study were that the NLR was significantly higher in non-surviving patients than in survivors and that plasma albumin was significantly lower in those who died than in survivors; both the NLR and albumin were independent predictors for 30-day mortality among patients with PAPE following noncardiac surgery. Moreover, the nomogram based on the NLR and albumin showed good performance in predicting 30-day mortality in patients with PAPE.

In our research, 101 patients were included, and 24 patients died within 30 days, corresponding to a 30-day mortality rate of 23.8%. According to previous studies, the mortality rate of APE patients ranges from 8.1–25.3% [12, 18–20]. Our results showed that the mortality of our cohort was within this range. In our research, the NLR was identified as an effective prognostic biomarker for PAPE patients. The NLR is the comprehensive presentation of systemic inflammation and the balance between neutrophils and lymphocytes in CBCs. Previous studies have shown that an elevated NLR is associated with an increased rate of hospital mortality among patients with acute pulmonary embolism [13], acute exacerbation of chronic obstructive pulmonary disease [21], and acute type A aortic dissection [22]; of 30-day mortality among patients with acute pulmonary embolism [19], acute kidney injury [23], ST-elevation myocardial infarction [24], and intracerebral hemorrhage [25, 26]; and of long-term mortality among patients with ST-elevation myocardial infarction [27], breast cancer [28] and epithelial ovarian cancer [29].

The link between inflammation and pulmonary embolism has been well investigated, although the underlying mechanism is not completely understood. The relationship between them may be linked by cytokines, proinflammatory cytokines, and acute-phase proteins, such as CRP, IL-8, and tumor necrosis factor, which promote the procoagulant state and play an important role in the progression of venous thromboembolism by inducing the expression of tissue factors. In addition, it has recently been reported that inflammatory mediators, such as polyphosphates and bradykinin, can directly activate contact systems and initiate external coagulation pathways [30–32]. In our research, we found that the NLR was an independent predictor of 30-day mortality in patients with PAPE, and the area under the curve of the NLR was 0.823. Therefore, we concluded that the NLR is a simple and effective prognostic predictor for patients with PAPE.

We also found that albumin was significantly lower in those who died than in survivors. To our knowledge, this is the first study to indicate the relationship between albumin and mortality in patients with PAPE. Albumin is an indicator of the nutritional status of patients and can regulate the anticoagulation system to some extent. Hypoproteinemia has been confirmed to be associated with mortality in patients with acute pulmonary embolism in previous studies [16]. In a previous study, the mechanism of the association between albumin and mortality was partly explained. Plasma albumin can interact with NO to some extent and generate S-nitrosoproteins, which then promote vasomotor activity and inhibit platelet aggregation. When albumin levels drop, the effect will be weakened [33, 34]. In addition, plasma albumin has important antioxidant, anti-inflammatory and drug carrier effects in human physiological functions [35]. Therefore, a lower plasma albumin concentration will inevitably lead to a decrease or loss of these effects.

There were also some limitations in our research. First, as a single-center study, only 101 patients met the criteria and were included in our study, which was a small sample size. The small sample size makes it impossible to classify and discuss patients with PAPE for specific operations, such as arthroplasty and gastrointestinal cancer resection. Second, although the nomogram showed good performance in terms of the AUC, calibration curve and DCA, independent validation is needed. Finally, as a retrospective study, our research had its own limitations, and some potential predictors were not included in our research. We hope that multicenter and prospective research can be performed to confirm our conclusion in the future.

## Conclusions

Both the NLR and albumin were independent predicators for 30-day mortality among patients with acute pulmonary embolism following noncardiac surgery, and the NLR and albumin were better predictors together than separately. This enables assessing the severity of PAPE and can guide the clinical management of PAPE.

## Data Availability

The datasets generated during and/or analyzed during the current study are available from the corresponding author on reasonable request.

## Abbreviations

PAPE: Postoperative acute pulmonary embolism
NLR: Neutrophil-to-lymphocyte ratio
MLR: Monocyte-to-lymphocyte ratio
RDW: Red blood cell distribution width
CRP: C-reactive protein\
CBC: Complete blood count
ROC: Receiver operating characteristic curve AUC Area under the curve
DCA: Decision curve analysis
LMR: Lymphocyte to monocyte ratio
